# Hydralazine-Induced Diarrhea in a Geriatric Patient: A Case Report and a Review of the Literature on Deprescribing as a Diagnostic Strategy

**DOI:** 10.7759/cureus.92359

**Published:** 2025-09-15

**Authors:** Azhar Hussain, Miriam Sprei, Leah Moradi

**Affiliations:** 1 Pharmaceutical and Biomedical Sciences, Touro University College of Pharmacy, New York, USA; 2 Internal Medicine, Touro University College of Pharmacy, New York, USA

**Keywords:** deprescribing, diarrhea, drug-induced diarrhea, hydralazine, naranjo algorithm

## Abstract

Older adults can be particularly susceptible to adverse drug reactions (ADRs) due to age-related physiological changes, decline in renal and hepatic function, and chronic comorbidities. Drug-induced diarrhea (DID) can often be hard to identify in the older adult population due to polypharmacy and the constant addition of new medications to treat the side effects of previous medications. The early identification of DID is important to prevent complications that can lead to prolonged hospitalization and increased mortality. Hydralazine, a vasodilator used for blood pressure management, can potentially cause gastrointestinal abnormalities such as nausea, vomiting, paralytic ileus, and constipation, but the frequency of these adverse effects is not specified. Here, we discuss a case of a 74-year-old hospitalized woman who developed diarrhea shortly after the initiation of hydralazine, which led to the development of an acute kidney injury and the need for prolonged hospitalization. The diarrhea worsened with hydralazine dose escalation and resolved within 24 hours of the discontinuation of hydralazine. Other medications that could potentially cause diarrhea, including antibiotics, intravenous magnesium, and HIV therapy, were ruled out by a thorough medication review. A score of 6 on the Naranjo algorithm indicates a probable cause relationship between the diarrhea and hydralazine. This case emphasizes the importance of thorough medication reviews to identify drugs that are not commonly associated with DID.

## Introduction

Age-related physiological changes, decline in renal and hepatic function, and chronic comorbidities often leave older adults particularly susceptible to adverse drug reactions (ADRs) such as diarrhea [1]. Drug-induced diarrhea (DID) is defined as the occurrence of loose or watery stools that can be temporally associated with the initiation of a new medication, typically within hours to several days, though onset may vary depending on the pharmacological properties of the agent involved. DID is attributed to the medication itself rather than an infectious or gastrointestinal cause [2]. The identification of DID in older adults is often challenging due to polypharmacy and the constant addition of new medications to treat the side effects of previous medications [2,3]. Mild cases of DID are often self-limiting. However, if DID is not identified and managed early in older adults, it can lead to serious complications such as dehydration, electrolyte imbalances, kidney injury, and arrhythmias, all of which are associated with increased mortality [4].

Hydralazine, a vasodilator used for blood pressure management, is not typically associated with DID, but diarrhea is listed as a common side effect in the prescribing information [5]. The mechanism by which hydralazine causes diarrhea is unknown, but it is possible that its systemic vasodilatory properties enhance gastrointestinal perfusion and motility, leading to diarrhea [4,6]. In hospitalized older adults, hydralazine may be added to the antihypertensive regimen when blood pressure remains uncontrolled despite other agents, especially in cases of resistant hypertension [5,6]. Here, we describe a case of suspected hydralazine-induced diarrhea in a hospitalized older adult, which worsened with a dose increase and resolved within 24 hours of stopping the medication. The purpose of this case report is to bring awareness to hydralazine as a potential cause of DID in older adults and the importance of a thorough medication review when evaluating new-onset gastrointestinal symptoms occurring shortly after starting a new medication.

## Case presentation

A 74-year-old woman with a past medical history of hypertension, HIV, stroke, chronic kidney disease, hypothyroidism, and tobacco and substance abuse was admitted to the emergency department following an unwitnessed fall. She had been found unresponsive with a loss of movement in her left arm, leading the medical team to suspect a stroke. While in the emergency department, her condition deteriorated, necessitating intubation for airway protection. On day 2 of hospitalization, the patient developed aspiration pneumonia and was started on empiric therapy. She received 2 g of ceftriaxone intravenously administered once daily for seven days, 100 mg of doxycycline administered orally twice daily for seven days, and 500 mg of metronidazole administered orally twice daily for seven days. The patient also received one dose of 500 mg of azithromycin orally on day 2 before being switched to doxycycline. No adverse effects or gastrointestinal complaints were reported after the initiation of antibiotics. After six days in the intensive care unit, the patient’s condition stabilized, so she was extubated and transferred to the neuroscience floor for continued monitoring.

**Figure 1:**
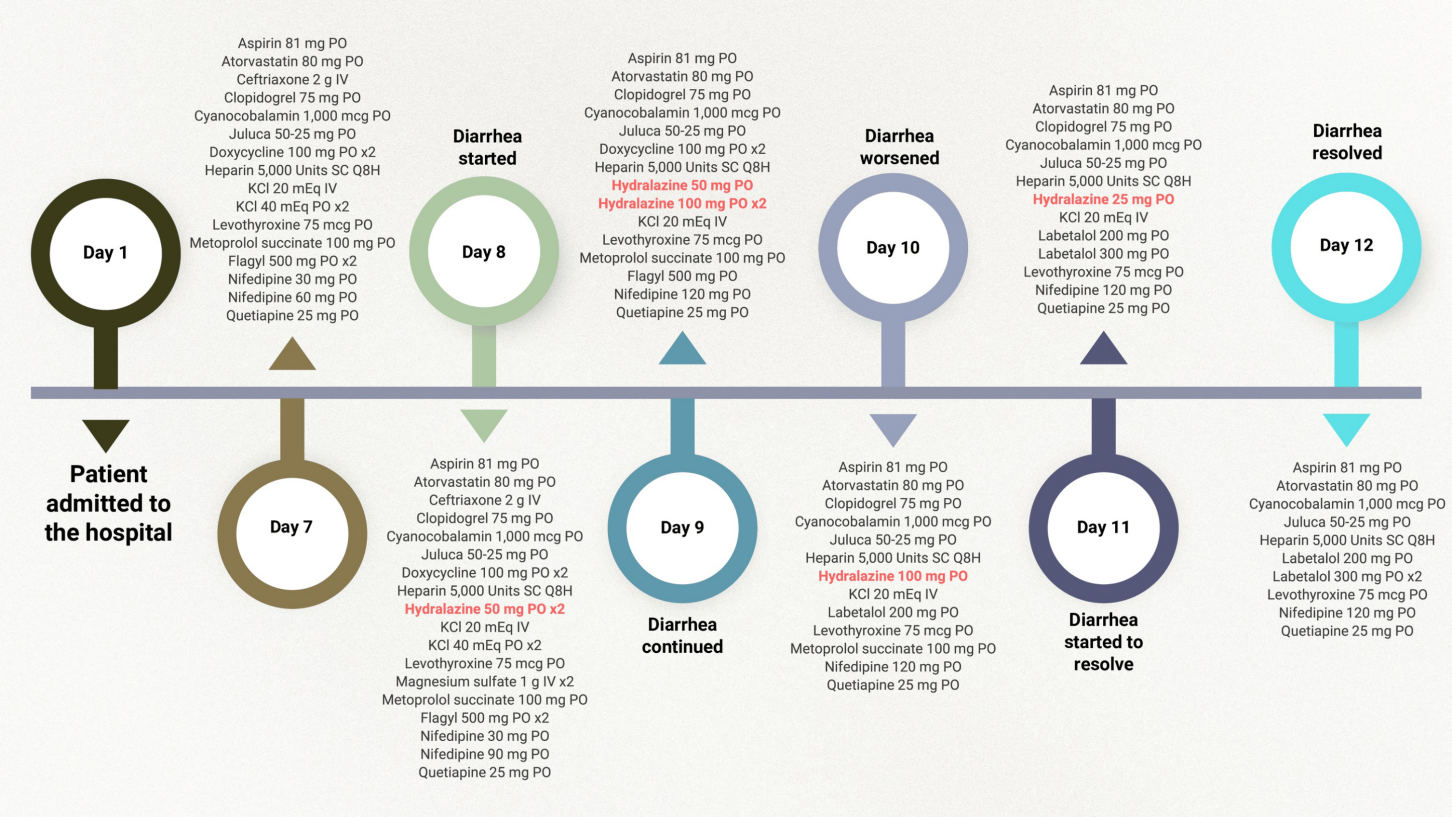
Patient medication timeline IV, intravenous; PO, by mouth (per os, oral administration); SC, subcutaneous; Q8H, every eight hours; x2, twice (usually refers to twice a day unless otherwise specified)

Throughout the time the patient was in the hospital, her blood pressure remained elevated (systolic blood pressure range of 150-170 mmHg) despite treatment with nifedipine. Therefore, hydralazine 50 mg administered orally twice a day was added on day 8 of hospitalization, as shown in Figure 1.

No improvement in blood pressure was observed on day 9, so the dose of hydralazine was increased to 100 mg twice daily. On day 10, after receiving a total of 100 mg of hydralazine on day 8, a total of 250 mg of hydralazine on day 9, and a total of 100 mg of hydralazine on day 10, the patient complained of several episodes of loose watery stool. She reported that the diarrhea had started two days prior (on day 8) and had worsened from day 9 to day 10 (after the hydralazine dose increase). The diarrhea caused the patient to develop an acute-on-chronic kidney injury, requiring intravenous fluids and increased laboratory evaluation, as shown in Table 1.

**Table 1:** Laboratory test results WBC, white blood cell; RBC, red blood cell; Hgb, hemoglobin; Hct, hematocrit; BUN, blood urea nitrogen; ALT, alanine aminotransferase; AST, aspartate aminotransferase; eGFR, estimated glomerular filtration rate

Laboratory Test	Day 1	Day 7	Day 8	Day 10	Day 11	Day 12	Day 13	Day 14	Reference Range
WBC	7.3	9.0	7.8	-	-	-	-	-	4.5-10.2 ×10^3^/μL
RBC	3.94	3.88	3.87	-	-	-	-	-	3.95-4.83 × 10^6^/μL
Hgb	11.9	11.7	11.9	-	-	-	-	-	11.4-15.5 g/dL
Hct	36.2	35.2	35.2	-	-	-	-	-	37%-43.7%
Platelets	248	282	286	-	-	-	-	-	180-401 × 10^3^/μL
Glucose	202	85	85	105	88	86	83	104	140-180 mg/dL
BUN	39	40	35	49	52	54	56	52	7-25 mg/dL
Creatinine	2.7	3.0	2.8	3.1	3.6	3.3	3.1	3.1	0.6-1.2 mg/dL
Sodium	138	143	143	139	143	143	142	142	136-145 mEq/L
Potassium	4.4	3.0	3.4	3.8	4.5	4.1	4.2	4.2	3.5-5.1 mEq/L
Chloride	105	107	106	111	113	115	115	115	98-107 mEq/L
Calcium	8.7	8.5	8.4	8.3	7.7	7.8	7.5	8.0	8.6-10.3 mg/dL
Phosphorus	6.9	2.1	2.6	-	-	-	-	-	2.5-5.0 mg/dL
Albumin	4.0	3.5	3.5	3.0	3.0	2.8	2.7	3.0	3.5-5.7 g/dL
ALT	72	34	31	21	19	17	17	17	7-52 U/L
AST	86	31	28	14	14	15	14	12	13-39 U/L
Alkaline phosphatase	86	78	80	57	69	71	79	80	34-104 U/L
eGFR	18	15.8	17.2	15.2	12.7	14.1	15.2	15.2	>90.0 mL/minute/1.73m^2^
Magnesium	2.1	2.0	1.7	1.7	-	-	-	-	1.9-2.7 mg/dL
Creatine kinase	239	531	475	-	-	-	-	-	30-223 U/L
Anion gap	12.0	11	10	7.0	10	8.00	9.00	7.0	8.00-12.00 mEq/L

The pharmacy team was consulted to evaluate the patient’s medications for a possible cause of her diarrhea. Following a thorough review, hydralazine was identified as a potential source. In response, the medical team decided to de-escalate hydralazine therapy to assess its impact on the patient’s diarrhea symptoms. On day 11, the patient received a single 25 mg dose of hydralazine. Following the decreased dose, the patient reported an improvement in her diarrhea. Given that the diarrhea improved when the dose of hydralazine was decreased, the medical team decided to stop hydralazine therapy altogether on day 11. By the following day (day 12), the patient reported the complete resolution of her diarrhea, prompting the medical team to conclude that hydralazine was the definite primary cause. Following improvement in the patient’s renal function, she was discharged from the hospital on day 17 on 81 mg aspirin once daily, 10 mg atorvastatin once daily, 100 mg gabapentin once daily, 50-25 mg Juluca once daily, 300 mg labetalol twice daily, 75 mcg levothyroxine once daily, 120 mg nifedipine once daily, 25 mg quetiapine once daily, and 1000 mcg vitamin B-12 once daily without any further complaints of diarrhea.

## Discussion

Although hydralazine’s prescribing information and major drug compendia, such as UpToDate, list diarrhea among its possible gastrointestinal adverse effects, the reported frequency is undefined, and to our knowledge, no prior case reports in the literature have specifically described hydralazine-induced diarrhea [7]. Most published accounts of hydralazine’s gastrointestinal toxicity focus on nausea, vomiting, or anorexia rather than diarrhea, highlighting the novelty of our observation. Hundreds of medications have been implicated in DID; however, hydralazine is not commonly considered one of them. In this case, the patient had been on several medications that could have potentially been the cause of her diarrhea, which is why it was necessary to do a thorough medication review to determine the most likely culprit.

Due to her aspiration pneumonia, the patient had been started on several antibiotics for seven days. Antibiotic-associated diarrhea is a common type of DID; however, in this case, it was ruled out for several reasons. DID typically develops within several hours to days of starting a new medication and resolves once the medication is stopped [8]. In this case, the diarrhea developed several days after the patient started taking antibiotics and worsened after the antibiotics were finished, making it unlikely that they were the cause of her diarrhea.

The patient also presented with hypomagnesemia, for which she received two doses of intravenous magnesium on day 8, and for HIV, she was taking Juluca as her home medication. Both of these medications are known to potentially cause diarrhea. However, the patient had received intravenous magnesium in the past without any complaints, and as with the antibiotics, the diarrhea worsened several days after the last dose of magnesium, which does not align with the typical presentation of DID. Similarly, the patient had been on Juluca for many years without any complaints of diarrhea, so it would be unlikely for her to start exhibiting that side effect now.

Since the other medications were ruled out as likely causes of her diarrhea, hydralazine remained the most probable culprit. According to the Naranjo algorithm, a scale used to assess the probability that an adverse drug reaction is related to a medication, the patient’s adverse reaction was assigned a score of 6, as shown in Table 2 [9]. This score suggests a probable cause between diarrhea and the use of hydralazine. Additionally, given that the timeline of diarrhea onset, worsening, and resolution aligned with the initiation, dose increase, de-escalation, and discontinuation of hydralazine, hydralazine was probably the contributing factor to this patient’s diarrhea.

**Table 2:** Adverse drug reaction probability scale (Naranjo algorithm) Score 0 or less, doubtful causality; scores 1-4, possible causality; scores 5-8, probable causality; scores >9, definite causality

Please Answer the Following Questions	Yes	No	Do Not Know	Score
1. Are there any previous reports from any source on this reaction indicating possible, probable, or definite relationship to drug/device/procedure (intervention) required by the protocol?	+1	0	0	0
2. Did the adverse event appear after the suspected drug/device/intervention was administered?	+2	-1	0	+2
3. Did the adverse reaction improve when the drug/device/intervention was discontinued or a specific antagonist was administered?	+1	0	0	+1
4. Did the adverse reaction reappear when the drug/device/intervention was readministered?	+2	-1	0	0
5. Are there alternative causes (other than the drug/device/intervention) that could, on their own, have caused the reaction?	-1	+2	0	+2
6. Did the reaction reappear when a placebo was given?	-1	+1	0	0
7. Was the drug/device detected in the blood (or other fluids) in concentrations known to be toxic?	+1	0	0	0
8. Was the reaction more severe when the dose was increased or less severe when the dose was decreased?	+1	0	0	+1
9. Did the patient have a similar reaction to the same or similar drug/device/intervention in any previous exposure?	+1	0	0	0
10. Was the adverse event confirmed by objective evidence?	+1	0	0	0
Total score				6

This case has several limitations. Stool studies, such as *Clostridioides difficile* testing and stool white blood cell testing, were not conducted, which poses a significant limitation as infectious and gastrointestinal causes for the diarrhea cannot be ruled out with complete certainty. Furthermore, there was no documentation regarding the frequency of bowel movements recorded by the nursing staff. All symptoms were reported by the patient. However, the onset of acute kidney injury on day 10, coinciding with the reported deterioration of diarrhea symptoms, indicates that the patient was undergoing considerable volume depletion, which is typical in cases of diarrhea. There were also several other medications that the patient was taking that are known to cause diarrhea and, therefore, represent potential confounders in this case. Lastly, a rechallenge test with hydralazine was not conducted to confirm that hydralazine was the definitive cause of the patient’s diarrhea. Despite these limitations, the timing of hydralazine initiation and the onset of diarrhea, the increase in hydralazine dosage and the worsening of diarrhea, and the subsequent discontinuation of hydralazine leading to the resolution of diarrhea support a strong case for hydralazine-induced diarrhea.

## Conclusions

This case identifies hydralazine as a potential cause of DID in older adults. Hydralazine is often added in the hospital for blood pressure management, and it is necessary to recognize that it can be associated with gastrointestinal side effects. Polypharmacy and chronic comorbidities pose a challenge in identifying DID, which is why thorough medication reviews are so important. Identifying DID early can help prevent complications such as diarrhea, electrolyte imbalances, kidney injury, or arrhythmias, which can prolong hospitalization and increase mortality. In this case, the patient experienced episodes of diarrhea shortly after the initiation of hydralazine, which worsened when the dose was increased and resolved when the dose was decreased, supporting a probable cause for hydralazine-induced diarrhea.
